# Energy transfer in Carreau Yasuda liquid influenced by engine oil with Magnetic dipole using tri-hybrid nanoparticles

**DOI:** 10.1038/s41598-023-32052-2

**Published:** 2023-04-03

**Authors:** Muhammad Bilal, Ikram Ullah, Mohammad Mahtab Alam, Syed Irfan Shah, Sayed M. Eldin

**Affiliations:** 1 Sheikh Taimur Academic Block-II, Department of Mathematics, University of Peshawar, Peshawar, 25120 Khyber Pakhtunkhwa Pakistan; 2 Department of Natural Sciences and Humanities, University of Engineering and Technology, Mardan, 23200 Pakistan; 3 Department of Basic Medical Sciences, College of Applied Medical Science, King Khalid University, Abha, 61421 Saudi Arabia; 4 Department of Sciences and Humanities, National University of Computer and Emerging Sciences, Islamabad, 44000 Pakistan; 5 Center of Research, Faculty of Engineering, Future University in Egypt, New Cairo, 11835 Egypt

**Keywords:** Engineering, Mathematics and computing, Nanoscience and technology

## Abstract

The aim of the current analysis is to evaluate the significances of magnetic dipole and heat transmission through ternary hybrid Carreau Yasuda nanoliquid flow across a vertical stretching sheet. The ternary compositions of Al_2_O_3_, SiO_2_, and TiO_2_ nanoparticles (nps) in the Carreau Yasuda fluid are used to prepare the ternary hybrid nanofluid (Thnf). The heat transfer and velocity are observed in context of heat source/sink and Darcy Forchhemier effect. Mathematically, the flow scenario has been expressed in form of the nonlinear system of PDEs for fluid velocity and energy propagation. The obtained set of PDEs are transform into ODEs through suitable replacements. The obtained dimensionless equations are computationally solved with the help of the parametric continuation method. It has been observed that the accumulation of Al_2_O_3_, SiO_2_ and TiO_2_-nps to the engine oil, improves the energy and momentum profiles. Furthermore, as compared to nanofluid and hybrid nanofluid, ternary hybrid nanofluid have a greater tendency to boost the thermal energy transfer. The fluid velocity lowers with the outcome of the ferrohydrodynamic interaction term, while enhances with the inclusion of nano particulates (Al_2_O_3_, SiO_2_ and TiO_2_).

## Introduction

The study of simple or hybrid nanofluid flow across a vertical surface, wither stretching or rigid plate with energy allocation characteristics has major commitment in recent developments and industrial uses^1^. Recently, Shah et al.^2^ documented the upshot of molecular diffusion on the flow characteristics of nanoliquid with concentration diffusivity and variable viscosity across a vertical sheet. It was revealed that greater temperature-dependent viscous factors improve velocity curve in both assisting and opposing flows. Chen et al.^3^ used computation algorithm to evaluate the fluid flows across a vertical surface, at Fr = 1.1 and Re = 2.7 105. Singh and Seth^4^ investigated the mass and thermal mobility behavior of MHD fluid flow inside a vertical stream bounded by the highly permeable regime using the Hall characteristic and an induced magnetic field. Shafiq et al.^5^ established a mathematical bioconvective model to analyze the thermodynamically thixotropic nanomaterials flow by implementing thermal radiation and convective conditions. The conclusions indicated that they can be utilised to improve heating and cooling procedures, manufacturing and energy generation among other factors. Fayz-Al-Asad et al.^6^ evaluated the MHD Maxwell fluid flow in conjunction with thermal conductivity and heat dependent viscosity along a stratified vertical surface using nth order fusion reaction. Sharma and Gandhi^7^ reviewed an unsteady MHD fluid flow across a vertical elongating surface implanted in a Darcy-Forchheimer permeable material with first-order chemical reaction and heat source/sink. Sharma et al.^8^ investigated an incompressible fluid flow across a variable vertical elongating sheet with additional effects of Ohmic heating, viscous dissipation, thermophoresis, thermal heat source, Brownian motion, activation energy and exponential heat source. The solar thermal transport features of hybrid nanoliquid in the existence of an external electromagnetic effect, radiation and heat source are investigated Rizk et al.^9^. Rooman et al.^10^ considered the enhancement of transfer of thermal energy in tri-hybrid Ellis nanoliquid flow when a magnetic polarization moves across a vertical substrate. It was discovered that the energy pattern advances with modification in heat generation and viscous dissipation. A magnetic dipole makes a substantial impact to the power generation, and an inverse correlation is indicated versus the flow pattern. Some recent analysis may be found in Ref.^11–14^.

Nanotechnology is an exciting scientific discipline with numerous implementations that range from skincare brands, groceries, apparels, and home electronics to fuel catalysts, therapeutic approaches, and alternative resources. Construction activities, nanomachining of nanostructures, nanowires, nanosheets, water purifiers, and waste management are all examples of how nanotechnology is being used in advanced manufacturing and detoxification operations^15–17^. Their implementations are expanding to include "nanomedicine" by fusing nanostructures with microbial macromolecules or frameworks, "green technology" to improve sustainable development, and "renewable energy" to establish new methods of capturing, storing, and transferring energy. Nanofiber generation, for example, has been used in applications like energy storage batteries, auto parts, thin-film telecommunications equipment, varnishes, and many more^18–20^. But such applications and uses of nanofluid in advanced techniques is only possible due to the involvement of hybrid and ternary nanofluid. Therefore, in the current analysis, we have used Al_2_O_3_, SiO_2_, and TiO_2_ in the engine oil. Okumura et al.^21^ used the Al_2_O_3_, TiO_2_ and SiO_2_, for the oxidation of H_2_ and CO for chemical vapor deposition of gold. Minea^22^ calculated the properties of oxide-based hybrid nanofluid (Al_2_O_3_, TiO_2_ and SiO_2_) and their derivatives. All type of nanoliquid' thermal performance changed with the inclusion of nanoparticles, and thermal expansion increased by at least 12%. Said et al.^23^ presented an experimental analysis on the density and stability of Al_2_O_3_, TiO_2_, TiSiO_4_ and SiO_2_. Minea^24^ Khan worked on a complex mathematical model on the energy transport efficiency and hydrostatic power of nanoliquids for a fluid dynamic assessment. The best flow behavior was observed when water was replaced with SiO_2_–Al_2_O_3_hybrid nanofluids. Abbasi et al.^25^ disclosed a correlative thermal evaluation of three sorts of nanoparticles, including aluminum oxide (Al_2_O_3_), titanium dioxide (TiO_2_) and silicon dioxide (SiO_2_) with the ethylene glycol base fluid over a circular cylinder containing the point of stagnation. Dadheech et al.^26^ reviewed the flow of an SiO2-Al2O3-TiO2/C2H6O2-based modified ferrofluid along an extending substrate. It was discovered that the thermal transfer capacity of modified nanoliquids is greater than that of simple and hybrid nanoliquids. Erkan et al.^27^ used Al_2_O_3_, TiO_2_, and SiO_2_ in ethylene glycol for engine radiator applications. As a result, using TiO_2_ particles yielded the highest energy conversion efficiency (35.67%). Alharbi et al.^28–30^ created a nanofluid model with TiO_2_ in the base fluid within a squeezing/dilating channel, which included nanoparticle accumulation effects and nonlinear thermal radiations. The outcome confirmed that the fluid mobility is substantially controlled by the high viscosity variation caused by nanomaterials aggregation. Furthermore, thermal radiations generate significant heat, which can be used to break down the accumulation of the nanomaterials. Some recent studies may be found in Ref.^31–34^.

A magnetic dipole is made up of two magnetic poles isolated by a small distance. A magnetic moment is a unit of measurement that signifies the magnetic strength and alignment of a magnet or other component that generates a magnetic field. The consequences of magnetic dipole on trihybrid nanoliquid flow are evaluated. Magnetic dipole fused with trihybrid nanoliquid performs an important function in energy transference^35^. The effectiveness of 2D Oldroyd-B fluid flow across a shrinking sheet with thermal buoyancy was highlighted by Bashir et al.^36^. The findings demonstrated that as the thermal relaxation time factor's value boosts, the proportion of heat transport lowers. Additionally, the rate of thermophoretic accumulation slows down as the thermophoretic index rises. To analyse the innovative fluid flow in the existence of magnetic dipoles, Shoaib et al.^37^ described the artificial neural network with Levenberg–Marquardt algorithm that is intelligence-based.

The objective of the current assessment is to evaluate the significances of magnetic dipole and heat transmission through ternary hybrid Carreau Yasuda nanoliquid flow across a vertical stretching sheet. The ternary compositions of Al_2_O_3_, SiO_2_, and TiO_2_-nps in the Carreau Yasuda fluid are used to prepare the ternary hybrid nanofluid (Thnf). The heat transfer and velocity are observed in context of heat source/sink and Darcy Forchhemier effect. Mathematically, the flow scenario has been expressed in form of the nonlinear system of PDEs for fluid velocity and energy propagation. The acquired set of PDEs are transform into ODEs through suitable substitutions. The obtained dimensionless equations are computationally solved with the help of the PCM. In the coming segment, the flow set-up has been verbalized, solved and discussed.

## Mathematical analysis

**Figure 1:**
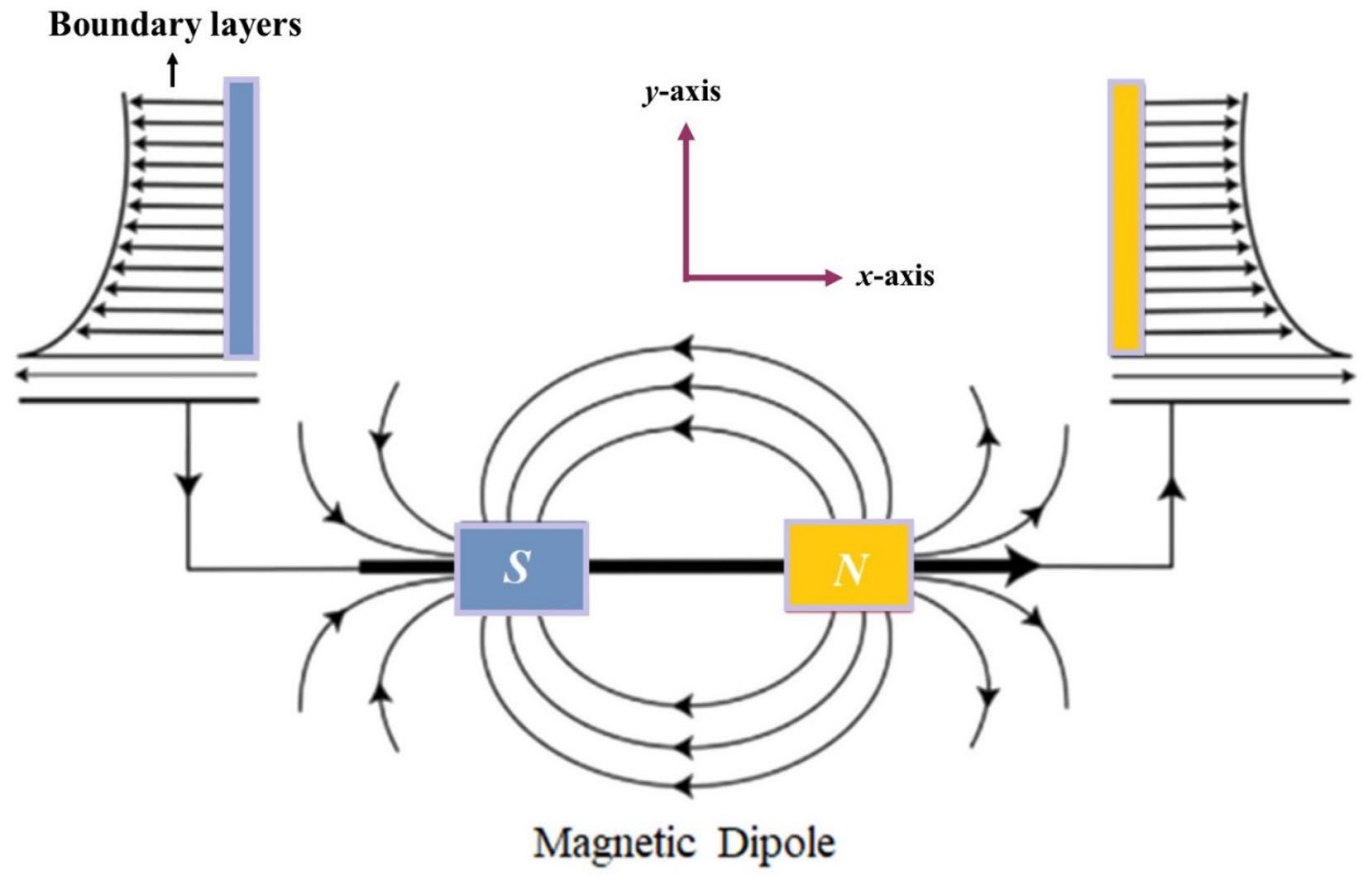
Fluid flow geometrical illustration.

The 2D Carreau Yasuda fluid with energy transfer is considered across an extending vertical sheet using ternary nanocomposites (TiO_2_, Al_2_O_3_ and SiO_2_). The Carreau Yasuda liquid is engrossed with base fluid (engine oil) in the Darcy medium. Three distinct kinds of nanocomposites (TiO_2_, Al_2_O_3_ and SiO_2_) are scattered in the base fluid. Surface of the wall is supposed to be stretchy to cause fluid motion. Hence, the fluid flow is due to the stretching of the sheet. Horizontally, the magnetic dipole is assumed to be in center as demonstrated in Fig. 1. The *x* & *y*-axis are taken in horizontal and vertical side of the sheet. The energy propagation is counted under the consequences of heat source. Based on the above suppositions, the modeled equations are formulated as^38,39^:

1 $$\frac{\partial u}{{\partial x}} + \frac{\partial v}{{\partial y}} = 0,$$

2 $$\rho_{Thnf} \left( {u\frac{\partial u}{{\partial x}} + v\frac{\partial u}{{\partial y}}} \right) = - \frac{\partial P}{{\partial x}} + \mu_{0} M\frac{\partial H}{{\partial x}} + \nu_{hnf} \left( {\frac{{\partial^{2} u}}{{\partial y^{2} }} + \Lambda^{d} \left( {\frac{m - 1}{d}} \right)\left( {d + 1} \right)\frac{{\partial^{2} u}}{{\partial y^{2} }}\left( {\frac{\partial u}{{\partial y}}} \right)^{d} } \right) - \frac{{\mu_{nf} }}{{\rho_{nf} K}}u - \frac{{c_{b} }}{\sqrt K }u^{2} ,$$

3 $$\left( {\rho C_{p} } \right)_{Thnf} \left( {u\frac{\partial T}{{\partial x}} + v\frac{\partial T}{{\partial y}}} \right) + \mu_{0} \left( {u\frac{\partial H}{{\partial x}} + v\frac{\partial H}{{\partial y}}} \right)T\frac{\partial M}{{\partial T}} = K_{Thnf} \frac{{\partial^{2} T}}{{\partial y^{2} }} + Q_{0} \left( {T - T_{\infty } } \right).$$

The boundary conditions are:

4 $$u = sx,\,\,\,v = 0,\,\,\,T = T_{w} ,\,\,\,u = 0,\,\,\,T = T_{\infty } .$$

The scalar potential with magnetic force is specified as:

5 $$\beta = \frac{\delta }{2\pi }\left( {\frac{x}{{x^{2} + \left( {y + d} \right)^{2} }}} \right).$$

*x*-axis and *y*-axis terms magnetic inductions are:

6 $$H_{x} = \frac{ - \partial \delta }{{\partial x}} = \frac{\delta }{2\pi }\left( {\frac{{x - \left( {d + y} \right)^{2} }}{{\left( {x^{2} + \left( {d + y} \right)^{2} } \right)^{2} }}} \right),$$

7 $$H_{y} = \frac{ - \partial \delta }{{\partial y}} = \frac{\delta }{2\pi }\left( {\frac{{2x\left( {d + y} \right)}}{{\left( {x^{2} + \left( {d + y} \right)^{2} } \right)^{2} }}} \right).$$

The magnitude of magnetic induction is

8 $$H = \left[ {\left( {\frac{\partial \delta }{{\partial y}}} \right)^{2} + \left( {\frac{\partial \delta }{{\partial x}}} \right)^{2} } \right]^{12} .$$

9 $$H_{b} = \frac{\partial \delta }{{\partial b}}\left( {\frac{ - 2}{{\left( {d + b} \right)^{3} }} + \frac{{4a^{2} }}{{\left( {d + b} \right)^{5} }}} \right),\,\,\,H_{a} = \frac{\partial \delta }{{\partial b}}\left( {\frac{ - 2a}{{\left( {d + b} \right)^{4} }}} \right).$$

The resemblance substitution is:

10 $$u = sxf^{\prime},\,\,\,v = - \left( {sv_{f} } \right)^{\frac{1}{2}} f,\,\,\,\theta = \frac{{T_{\infty } - T}}{{T_{\infty } - T_{w} }},\,\,\,\eta = y\sqrt{\frac{s}{y}}$$

The obtained dimensionless system of ODEs is:

11 $$f^{\prime\prime\prime} + \left( {We} \right)^{d} \frac{{\left( {m - 1} \right)\left( {d + 1} \right)}}{d}f^{\prime\prime\prime}\left( {f^{\prime\prime}} \right)^{d} - \frac{{v_{Thnf} }}{{v_{f} }}\left( {f^{\prime 2} - ff^{\prime\prime} + \frac{{2\beta \theta \rho_{f} }}{{\left( {\eta + \gamma } \right)^{4} }}} \right) - Fr\left( {f^{\prime}} \right)^{2} - kr{\mkern 1mu} f^{\prime } .$$

12 $$\begin{gathered} \theta^{\prime\prime} + \frac{{\left( {\rho C_{p} } \right)_{Thnf} k_{f} }}{{\left( {\rho C_{p} } \right)_{f} k_{Thnf} }}Pr\left( {f\theta^{\prime} - 2f^{\prime}\theta } \right) + \frac{{\left( {\rho C_{p} } \right)_{Thnf} k_{f} }}{{\left( {\rho C_{p} } \right)_{f} k_{Thnf} }}\frac{{2\beta \lambda f\left( {\theta - \varepsilon } \right)}}{{\left( {\eta + \gamma } \right)^{3} }} + \frac{{k_{f} \left( {PrH_{t} \theta } \right)}}{{k_{Thnf} }} - \frac{{k_{f} }}{{k_{Thnf} }} \hfill \\ \frac{{4\lambda \left( {1 - \phi_{2} } \right)^{ - 2.5} }}{{\left( {1 - \phi_{1} } \right)^{2.5} \left( {1 - \phi_{3} } \right)^{2.5} }}\left( {f^{\prime\prime}} \right)^{2} = 0, \hfill \\ \end{gathered}$$

The transform boundary conditions are:

13 $$\begin{gathered} f\left( 0 \right) = 0,\,\,\,\,\,\theta \left( 0 \right) = 1,\,\,\,\,f^{\prime}\left( 0 \right) = 1, \hfill \\ f^{\prime}\left( \infty \right) = 0,\,\,\theta \left( \infty \right) = 0. \hfill \\ \end{gathered}$$

The non- dimensional parameters are: $\beta = \frac{\gamma }{2\pi }\frac{\mu_{0} (T_{\infty } - T_{w})\rho}{{\mu}^{2}},  Pr = \frac{\nu} {\alpha}, \varepsilon = \frac{{T_{\infty }}}{{T_{\infty } - T_{w} }}, \lambda = \frac{{su}}{\rho{K} (T_{\infty } - T_{w} )}, \gamma = \sqrt{\frac {s\rho{c}^{2}}{{\mu}}}, H_{t} = \frac {Q_{0}} {b( {\rho C_{p} })_{bf}}$. It is observed that Eq. (11), (12) are non-Newtonian fluid model. The non-Newtonian model may be simplified to Newtonian case by putting $\beta = 0$ and *We* = 0.

The skin friction is expressed as:

14 $$\sqrt {Re} C_{f} = \frac{{ - \left( {\frac{m - 1}{d}We^{2} \left( {f^{\prime\prime}\left( 0 \right)} \right)^{d} + 1} \right)f^{\prime\prime}\left( 0 \right)}}{{\left( {1 - \phi_{3} } \right)^{2.5} \left( {1 - \phi_{2} } \right)^{2.5} \left( {1 - \phi_{1} } \right)^{2.5} }},$$

The Nusselt number is expressed as:

15 $$\left( {Re} \right)^{{\frac{ - 1}{2}}} Nu = \frac{{ - K_{f} }}{{K_{Thnf} }}\theta^{\prime}\left( 0 \right)$$

## Numerical solution

The core steps, while solving Eqs. (11)–(13) through parametric continuation method are as follow^41–43^:

### Step 1: reducing the system of BVP to 1st order

16 $$q_{1} = f(\eta ),\,\,\,q_{2} = f^{\prime}(\eta ),\,\,\,q_{3} = f^{\prime\prime}(\eta ),\,\,\,\,q_{4} = \theta (\eta ),\,\,\,q_{5} = \theta^{\prime}(\eta ).$$

By using Eq. (16) in Eqs. (11)–(13), we get:

17 $$q^{\prime}_{3} + \left( {We} \right)^{d} \frac{{\left( {m - 1} \right)\left( {d + 1} \right)}}{d}q^{\prime}_{3} \left( {q_{3} } \right)^{d} - \frac{{v_{Thnf} }}{{v_{f} }}\left( {q_{2}^{2} - q_{1} q_{3} + \frac{{2\beta q_{4} \rho_{f} }}{{\left( {\eta + \gamma } \right)^{4} }}} \right) - Fr\left( {q_{2} } \right)^{2} - kr\,q_{2} .$$

18 $$\begin{gathered} q^{\prime}_{5} + \frac{{\left( {\rho C_{p} } \right)_{Thnf} k_{f} }}{{\left( {\rho C_{p} } \right)_{f} k_{Thnf} }}Pr\left( {q_{1} q_{5} - 2q_{2} q_{4} } \right) + \frac{{\left( {\rho C_{p} } \right)_{Thnf} k_{f} }}{{\left( {\rho C_{p} } \right)_{f} k_{Thnf} }}\frac{{2\beta \lambda q_{1} \left( {q_{4} - \varepsilon } \right)}}{{\left( {\eta + \gamma } \right)^{3} }} + \frac{{k_{f} \left( {PrH_{t} q_{4} } \right)}}{{k_{Thnf} }} \hfill \\ - \frac{{k_{f} }}{{k_{Thnf} }}\frac{{4\lambda \left( {1 - \phi_{2} } \right)^{ - 2.5} }}{{\left( {1 - \phi_{1} } \right)^{2.5} \left( {1 - \phi_{3} } \right)^{2.5} }}\left( {q_{3} } \right)^{2} = 0, \hfill \\ \end{gathered}$$

with the corresponding boundary conditions

19 $$\begin{gathered} q_{1} \left( 0 \right) = 0,\,\,\,q_{2} \left( 0 \right) = 1,\,\,\,q_{4} \left( 0 \right) = 1, \hfill \\ q_{2} \left( \infty \right) = 0,\,\,\,q_{4} \left( \infty \right) = 0. \hfill \\ \end{gathered}$$

### Step 2: presenting the embedding constraint p in Eqs. (17)–(19)

20 $$q^{\prime}_{3} + \left( {We} \right)^{d} \frac{{\left( {m - 1} \right)\left( {d + 1} \right)}}{d}q^{\prime}_{3} \left( {q_{3} - 1} \right)^{d} p - \frac{{v_{Thnf} }}{{v_{f} }}\left( {q_{2}^{2} - q_{1} q_{3} + \frac{{2\beta q_{4} \rho_{f} }}{{\left( {\eta + \gamma } \right)^{4} }}} \right) - Fr\left( {q_{2} } \right)^{2} - kr\,q_{2} .$$

21 $$\begin{gathered} q^{\prime}_{5} + \frac{{\left( {\rho C_{p} } \right)_{Thnf} k_{f} }}{{\left( {\rho C_{p} } \right)_{f} k_{Thnf} }}Pr\left( {q_{1} \left( {q_{5} - 1} \right)p - 2q_{2} q_{4} } \right) + \frac{{\left( {\rho C_{p} } \right)_{Thnf} k_{f} }}{{\left( {\rho C_{p} } \right)_{f} k_{Thnf} }}\frac{{2\beta \lambda q_{1} \left( {q_{4} - \varepsilon } \right)}}{{\left( {\eta + \gamma } \right)^{3} }} + \frac{{k_{f} \left( {PrH_{t} q_{4} } \right)}}{{k_{Thnf} }} \hfill \\ - \frac{{k_{f} }}{{k_{Thnf} }}\frac{{4\lambda \left( {1 - \phi_{2} } \right)^{ - 2.5} }}{{\left( {1 - \phi_{1} } \right)^{2.5} \left( {1 - \phi_{3} } \right)^{2.5} }}\left( {q_{3} } \right)^{2} = 0, \hfill \\ \end{gathered}$$

## Results and discussion

The trend physical process and behind each Table and figure are elaborated in this section. This section also revealed the velocity $f^{\prime}\left( \eta \right)$ and energy $\theta \left( \eta \right)$ profiles outlines versus physical constraints**.**

**Figure 2:**
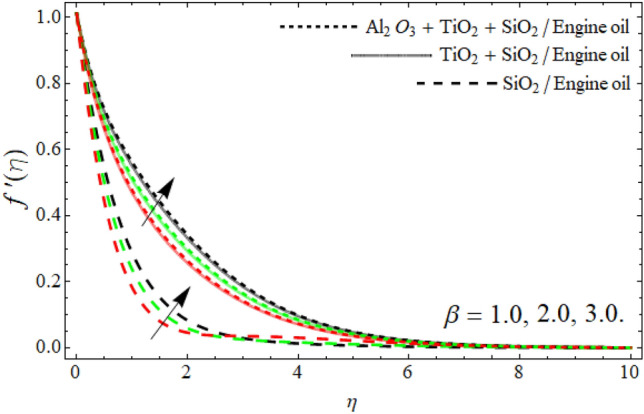
Velocity $f^{\prime}\left( \eta \right)$ field versus ferrohydrodynamic interaction number $\beta .$

**Figure 3:**
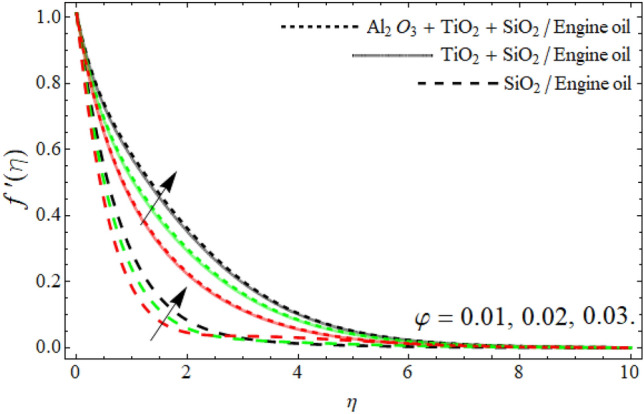
Velocity $f^{\prime}\left( \eta \right)$ field versus ternary nanoparticles $\varphi .$

**Figure 4:**
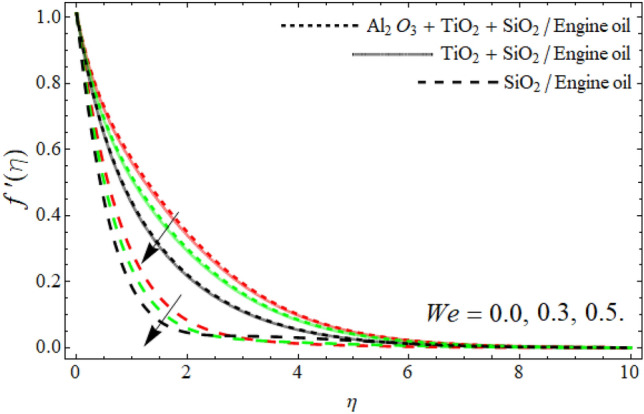
Velocity $f^{\prime}\left( \eta \right)$ field versus Weissenberg number *We*.

**Figure 5:**
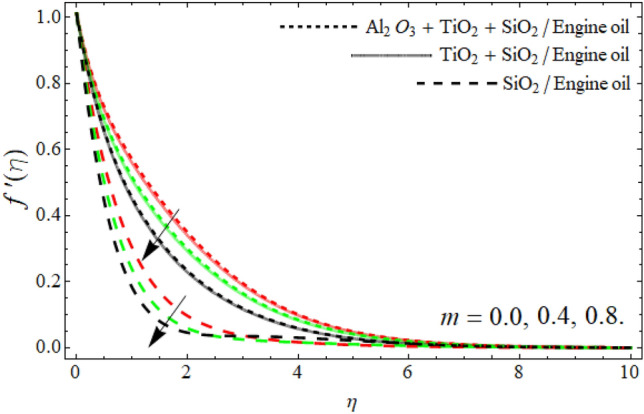
Velocity $f^{\prime}\left( \eta \right)$ field versus power law number *m*.

**Figure 6:**
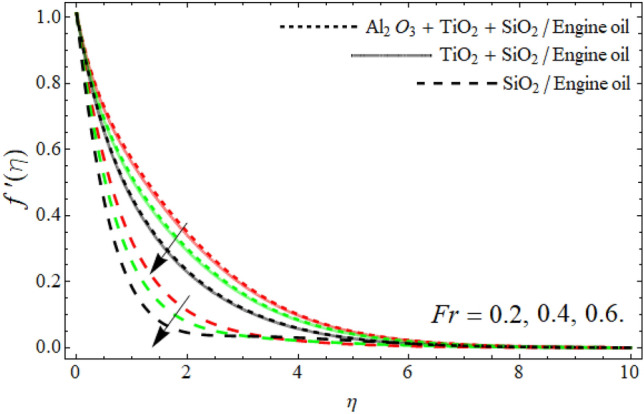
Velocity $f^{\prime}\left( \eta \right)$ field versus *Fr*.

**Figure 7:**
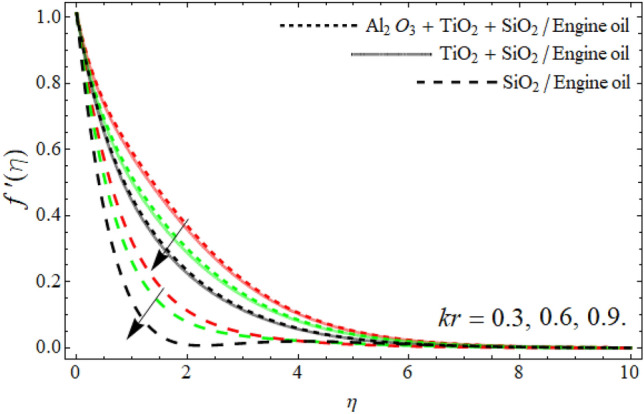
Velocity $f^{\prime}\left( \eta \right)$ field versus porosity term *kr*.

Figures 2, 3, 4, 5, 6, 7 display the velocity profile $f^{\prime}\left( \eta \right)$ outlines versus ferrohydrodynamic interaction number $\beta ,$ ternary nanoparticles $\varphi \left( \eta \right),$ Weissenberg number *We*, power-law number *m*, Darcy Forchhemier term *Fr* and porosity term *kr* respectively. The variance in velocity profile versus the significance $\beta$ is depicted in Fig. 2. It has been observed that a magnetic dipole draws fluid molecules at the wall's surface and that this pulling of fluid droplets toward the magnetic dipole causes friction between layers and particles. Hence, the velocity of fluid particles slows down. As a result, the fact that velocity curves have a decreasing function against the consequences $\beta ,$ is incorporated. The graph is examined in both the absence of a dipole and the presence of a magnetic dipole.

Figure 3 reported that the inclusion of nano particulates in the base fluid augments the momentum profile. The density of engine oil as compared to Al_2_O_3_, SiO_2_ and TiO_2_ is much higher. Therefore, the insertion of these nanoparticles into the engine oil reduces its average density, as a result, the velocity contour enhances. Figures 4 and 5 show that the velocity $f^{\prime}\left( \eta \right)$ declines with the upshot of *We* and power-law number *m*. A Weissenberg number is a physical ratio between elastic and viscous forces. It can be shown that an increase in *We* leads to an upsurge in the viscosity of fluid particles. As a result, the fluid becomes much thicker and reduces the number of layers of momentum boundary as elaborated in Fig. 4.

The power-law component is designed to evaluate the fluid category's behavior between layers. It's worth noting that m is a non-dimensional quantity generated as a result of the Carreau Yasuda fluid. When *m* is raised, the velocity curve shrinkages. Frictional forces are formed between momentum layers, and frictional forces cause fluid to thicken as shown in Fig. 5. Figures 6 and 7 revealed that the velocity outlines diminish with the upshot of Darcy Forchheimer’s term *Fr* and porosity term. The rising effect of Darcy and porosity term enhances with the porosity of the vertical stretching surface, which resists the fluid flow, as result, the velocity field drops.

**Figure 8:**
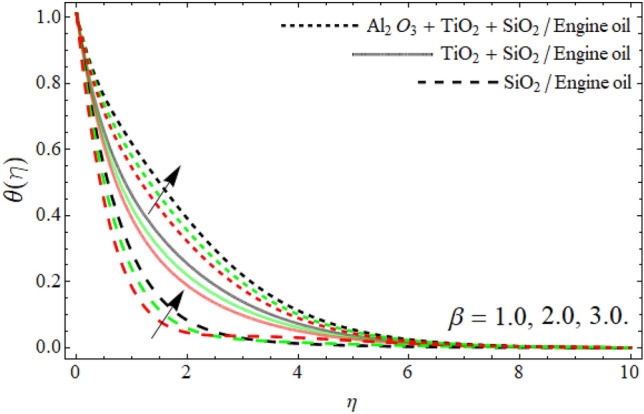
The energy $\theta \left( \eta \right)$ outlines versus ferrohydrodynamic interaction number $\beta .$

**Figure 9:**
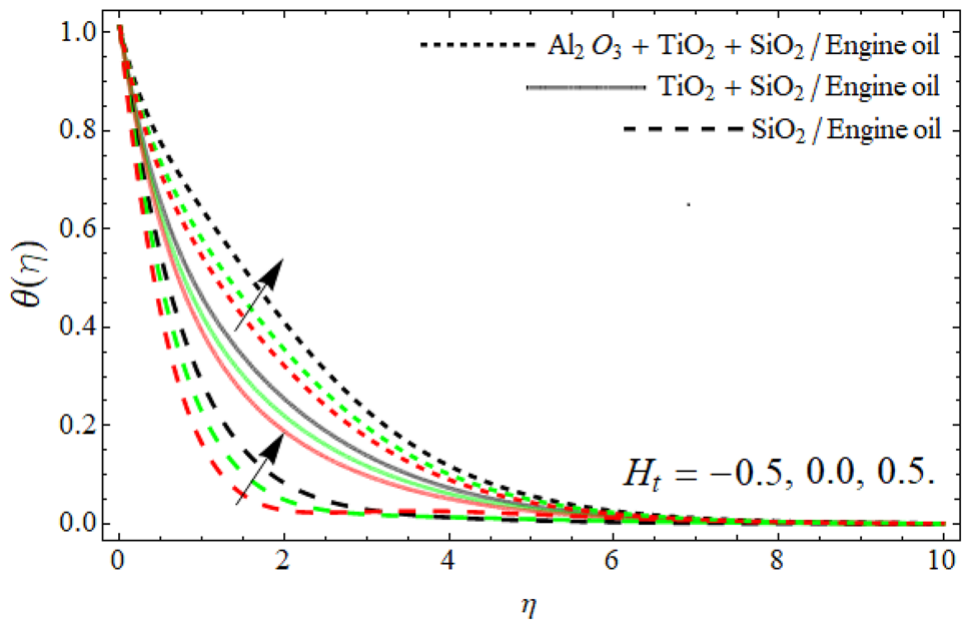
The energy $\theta \left( \eta \right)$ outlines versus *H*_*t*_.

**Figure 10:**
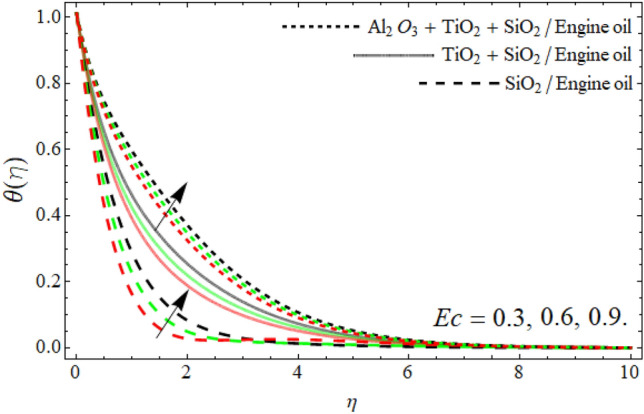
The energy $\theta \left( \eta \right)$ outlines versus Eckert number *Ec*.

**Figure 11:**
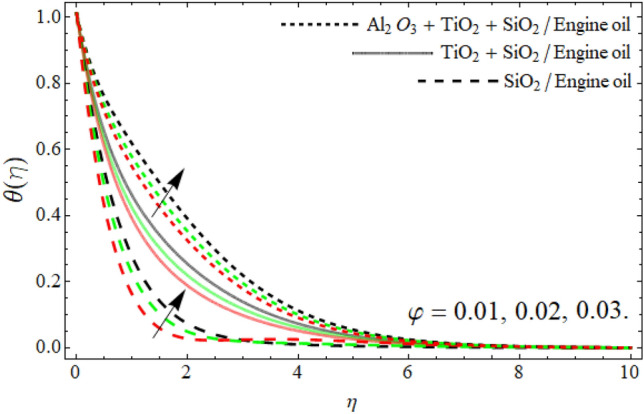
The energy profile $\theta \left( \eta \right)$ outlines versus ternary nanoparticles $\varphi .$

Figures 8, 9, 10, 11 exposed the nature of energy $\theta \left( \eta \right)$ curve versus the variation of ferrohydrodynamic interaction number $\beta ,$ heat source term *H*_*t*_, Eckert number *Ec* and ternary nanoparticles $\varphi$ respectively. It can be perceived that the upshot of all parameters $\beta ,$* H*_*t*_, *Ec* and $\varphi$ significantly boosts the energy profile. The magnetic dipole draws fluid molecules at the wall's surface, and this pulling of fluid droplets toward the magnetic dipole causes friction between layers and particles. Hence, the energy profile of ternary nanofluid enhances as depicted in Fig. 8. The influence of heat source term and Eckert number results in the additional heat inside the fluid flow, which causes the inclination of the temperature field as shown in Figs. 9 and 10. Figure 11 reported that the inclusion of nano particulates in the base fluid amplifies the energy profile. The density of engine oil as compared to Al_2_O_3_, SiO_2_ and TiO_2_ is much higher. Therefore, the insertion of these nanoparticles into the engine oil reduces its average density. On the other hand, the thermal conductivity of trihybrid nanoparticles is greater than base fluid, that’s why, the dispersion of the nano particulates, enhances the thermal profile of trihybrid nanofluids as shown in Fig. 11.

Tables 1 and 2 demonstrate the experimental values of ternary hybrid nanoparticles and engine oil and the basic mathematical model used for the simulation of trihybrid nanofluid flow. The consequences of skin friction and Nusselt number on Weissenberg number, viscous dissipation, heat source and power-law term are plotted in Table 3. Table 3 shows that greater numeric quantities of the heat source factor result in declination in heat transfer and flow rate. When the Weissenberg number is elevated, however, the flow rate improves. The importance of the power-law number in developing the highest quantity of heat transference rate and the flow rate has been noted.

**Table 1:** The investigational values of Al_2_O_3_, SiO_2_, TiO_2_ and engine oil^40^.

	*k*	$\sigma$	$\rho$
Engine oil	0.144	0.125 × 10^−11^	884
Al_2_O_3_	32.9	5.96 × 10^7^	6.310
TiO_2_	8.953	2.4 × 10^6^	4.250
SiO_2_	1.4013	3.5 × 10^6^	2.270

**Table 2:** The thermal properties of trihybrid nanoliquids^40^.

Viscosity	$\frac{{\mu_{Thnf} }}{{\mu_{f} }} = \frac{1}{{(1 - \phi_{{{\text{Al}}_{2} {\text{O}}_{3} }} )^{2.5} (1 - \phi_{{{\text{TiO}}_{2} }} )^{2.5} (1 - \phi_{{{\text{SiO}}_{2} }} )^{2.5} }},$
Density	$\frac{{\rho_{Thnf} }}{{\rho_{f} }} = \left( {1 - \phi_{{{\text{TiO}}_{2} }} } \right)\left[ {\left( {1 - \phi_{{{\text{TiO}}_{2} }} } \right)\left\{ {\left( {1 - \phi_{{{\text{SiO}}_{2} }} } \right) + \phi_{{{\text{SiO}}_{2} }} \frac{{\rho_{{{\text{SiO}}_{2} }} }}{{\rho_{f} }}} \right\} + \phi_{{TiO_{2} }} \frac{{\rho_{{{\text{TiO}}_{2} }} }}{{\rho_{f} }}} \right] + \phi_{{Al_{2} O_{3} }} \frac{{\rho_{{{\text{Al}}_{2} {\text{O}}_{3} }} }}{{\rho_{f} }},$
Specific heat	$\left. {\frac{{(\rho cp)_{Thnf} }}{{\left( {\rho cp} \right)_{f} }} = \phi_{{Al_{2} O_{3} }} \frac{{\left( {\rho cp} \right)_{{{\text{Al}}_{2} {\text{O}}_{3} }} }}{{\left( {\rho cp} \right)_{f} }} + \left( {1 - \phi_{{{\text{Al}}_{2} {\text{O}}_{3} }} } \right)\left[ {\left( {1 - \phi_{{{\text{TiO}}_{2} }} } \right)\left\{ {\left( {1 - \phi_{{{\text{SiO}}_{2} }} } \right) + \phi_{{{\text{SiO}}_{2} }} \frac{{\left( {\rho cp} \right)_{{{\text{SiO}}_{2} }} }}{{\left( {\rho cp} \right)_{f} }}} \right\} + \phi_{{{\text{TiO}}_{2} }} \frac{{\left( {\rho cp} \right)_{{{\text{TiO}}_{2} }} }}{{\left( {\rho cp} \right)_{f} }}} \right]} \right\}$
Thermal conduction	$$\left. \begin{gathered} \frac{{k_{Thnf} }}{{k_{hnf} }} = \left( {\frac{{k_{{{\text{SiO}}_{2} }} + 2k_{hnf} - 2\phi_{{{\text{SiO}}_{2} }} \left( {k_{hnf} - k_{{{\text{SiO}}_{2} }} } \right)}}{{k_{{{\text{SiO}}_{2} }} + 2k_{hnf} + \phi_{{{\text{SiO}}_{2} }} \left( {k_{hnf} - k_{{{\text{SiO}}_{2} }} } \right)}}} \right),\frac{{k_{hnf} }}{{k_{nf} }} = \left( {\frac{{k_{{{\text{TiO}}_{2} }} + 2k_{nf} - 2\phi_{{{\text{TiO}}_{2} }} \left( {k_{nf} - k_{{{\text{TiO}}_{2} }} } \right)}}{{k_{{{\text{TiO}}_{2} }} + 2k_{nf} + \phi_{{{\text{TiO}}_{2} }} \left( {k_{nf} - k_{{{\text{TiO}}_{2} }} } \right)}}} \right), \hfill \\ \frac{{k_{nf} }}{{k_{f} }} = \left( {\frac{{k_{{{\text{Al}}_{2} {\text{O}}_{3} }} + 2k_{f} - 2\phi_{{{\text{Al}}_{2} {\text{O}}_{3} }} \left( {k_{f} - k_{{{\text{Al}}_{2} {\text{O}}_{3} }} } \right)}}{{k_{{{\text{Al}}_{2} {\text{O}}_{3} }} + 2k_{f} + \phi_{{{\text{Al}}_{2} {\text{O}}_{3} }} \left( {k_{f} - k_{{{\text{Al}}_{2} {\text{O}}_{3} }} } \right)}}} \right), \hfill \\ \end{gathered} \right\}$$
Electrical conductivity	$$\begin{gathered} \frac{{\sigma_{Thnf} }}{{\sigma_{hnf} }} = \left( {1 + \frac{{3\left( {\frac{{\sigma_{{{\text{SiO}}_{2} }} }}{{\sigma_{hnf} }} - 1} \right)\phi_{{SiO_{2} }} }}{{\left( {\frac{{\sigma_{{{\text{SiO}}_{2} }} }}{{\sigma_{hnf} }} + 2} \right) - \left( {\frac{{\sigma_{{{\text{SiO}}_{2} }} }}{{\sigma_{hnf} }} - 1} \right)\phi_{{{\text{SiO}}_{2} }} }}} \right),\,\frac{{\sigma_{hnf} }}{{\sigma_{nf} }} = \left( {1 + \frac{{3\left( {\frac{{\sigma_{{{\text{TiO}}_{2} }} }}{{\sigma_{nf} }} - 1} \right)\phi_{{TiO_{2} }} }}{{\left( {\frac{{\sigma_{{{\text{TiO}}_{2} }} }}{{\sigma_{nf} }} + 2} \right) - \left( {\frac{{\sigma_{{{\text{TiO}}_{2} }} }}{{\sigma_{nf} }} - 1} \right)\phi_{{{\text{TiO}}_{2} }} }}} \right), \hfill \\ \frac{{\sigma_{nf} }}{{\sigma_{f} }} = \left( {1 + \frac{{3\left( {\frac{{\sigma_{{{\text{Al}}_{2} {\text{O}}_{3} }} }}{{\sigma_{f} }} - 1} \right)\phi_{{{\text{Al}}_{2} {\text{O}}_{3} }} }}{{\left( {\frac{{\sigma_{{{\text{Al}}_{2} {\text{O}}_{3} }} }}{{\sigma_{f} }} + 2} \right) - \left( {\frac{{\sigma_{{{\text{Al}}_{2} {\text{O}}_{3} }} }}{{\sigma_{f} }} - 1} \right)\phi_{{{\text{Al}}_{2} {\text{O}}_{3} }} }}} \right). \hfill \\ \end{gathered}$$

**Table 3:** The statistical outputs of skin friction $- \left( {Re} \right)^{\frac{1}{2}} C_{f}$ and Nusselt number $- \left( {Re} \right)^{{ - \frac{1}{2}}} Nu$ as well as its comparison with the existing literature.

Parameters	Values	$- \left( {Re} \right)^{\frac{1}{2}} C_{f}$^44^	$- \left( {\text{Re}} \right)^{\frac{1}{2}} C_{f}$	$- \left( {Re} \right)^{{ - \frac{1}{2}}} Nu$^44^	$- \left( {Re} \right)^{{ - \frac{1}{2}}} Nu$
	0.0	0.2620880941	0.2620880862	0.6269013682	0.6269013784
*We*	0.5	0.2805115031	0.2805115133	0.6150054882	0.6150054981
	1.5	0.2983830010	0.2983830212	0.6038359770	0.6038359872
	− 1.5	0.4793858421	0.47938585213	0.3524512521	0.3524512623
*H*_*t*_	0.0	0.3864732265	0.3864732366	0.2572567431	0.2572567533
	0.7	0.1464269369	0.1464269465	0.2116717874	0.2116717775
	0.1	0.0746743038	0.0746743237	0.3641187594	0.3641187793
*m*	0.4	0.1547000729	0.1547000828	0.5585378446	0.5585378647
	0.7	0.2398563119	0.2398563218	0.7083750632	0.7083750835
	0.0	0.2619120138	0.2619120339	0.7417200656	0.7417200755
$\lambda$	0.4	0.2614988160	0.2614988261	0.6310408286	0.6310408484
	0.8	0.2612510196	0.2612510397	0.5406325980	0.5406326871

## Conclusion

We have studied the significances of magnetic dipole and heat transmission through ternary hybrid Carreau Yasuda nanoliquid flow across a vertical stretching sheet. The ternary compositions of Al_2_O_3_, SiO_2_, and TiO_2_-nps in the Carreau Yasuda fluid are used to prepare the Thnf. The heat transfer and velocity are observed in context of heat source/sink and Darcy Forchhemier effect. Mathematically, the flow scenario has been expressed in form of the nonlinear system of PDEs for fluid velocity and energy propagation. The obtained set of PDEs are transform into ODEs through suitable substitutions. The obtained dimensionless equations are computationally solved with the help of the PCM. The main outcomes are:

- The accumulation of Al_2_O_3_, SiO_2_ and TiO_2_-nps to the engine oil, advances the energy and momentum profiles.
- Relative to simple fluid, ternary hybrid nanofluid have a greater tendency to boost the energy transmission across a vertical plate.
- The fluid velocity $f^{\prime}\left( \eta \right)$ lowers with the outcome of the ferrohydrodynamic interaction term, while enhances with the inclusion of nano particulates (Al_2_O_3_, SiO_2_ and TiO_2_) in the base fluid.
- The fluid velocity contour declines with the upshot of *We* and power-law number *m*.
- The escalating influence of Darcy Forchheimer’s term and porosity constant reduces the velocity outlines.
- The energy contour $\theta \left( \eta \right)$ enhances with the variation of ferrohydrodynamic interaction number, heat source term, Eckert number and ternary nanoparticles.
- The rising effects of the power-law index remarkably elevate the skin friction and Nusselt number of trihybrids nanofluid.

## Data Availability

All data used in this manuscript have been presented within the article.

## List of symbols

$$v,\,u$$: Velocity components
$$\mu_{0}$$: Magnetic permeability
$$C_{p}$$: Specific heat
*T*: Temperature
$$\pi$$: *Pi*
$$\sigma$$: Electrical conductivity
nps: Nanoparticles
*p*: Pressure
*k*: Thermal conductivity
$$T_{w}$$: Wall temperature
*Nu*: Nusselt number
*M*: Magnetization
*H*_*t*_: Heat source
$$\theta \left( \eta \right)$$: Energy field
$$\beta$$: Ferrohydrodynamic term
*We*: Weissenberg number
Al_2_O_3_: Aluminum oxide
*Ec*: Eckert number
$$\rho$$: Density
$\Lambda$: Carreau Yasuda number
$$Q_{0}$$: Heat source
$u_{e}$: Free stream velocity
$$\lambda$$: Viscous dissipation
*Re*: Reynolds number
Thnf: Trihybrid nanofluid
$\gamma$: Strength of the magnetic dipole
$S_{1}$: Stretching ratio number
$$\varepsilon$$: Ratio parameter
$$C_{f}$$: Skin fraction
*Pr*: Prandtl number
$\varphi$: Nanoparticles volume friction
$$f^{\prime}\left( \eta \right)$$: Velocity profile
*Fr*: Darcy Forchhemier term
*m*: Power-law number
SiO_2_: Silicon dioxide
TiO_2_: Titanium dioxide
